# Quantification of left ventricular trabeculae using fractal analysis

**DOI:** 10.1186/1532-429X-15-36

**Published:** 2013-05-10

**Authors:** Gabriella Captur, Vivek Muthurangu, Christopher Cook, Andrew S Flett, Robert Wilson, Andrea Barison, Daniel M Sado, Sarah Anderson, William J McKenna, Timothy J Mohun, Perry M Elliott, James C Moon

**Affiliations:** 1 Division of Cardiovascular Imaging, The Heart Hospital, part of University College London NHS Foundation Trust, 16-18 Westmoreland Street, London, W1G 8PH, UK; 2 UCL Institute of Cardiovascular Science, University College London, Gower Street, London, WC1E 6BT, UK; 3 UCL Centre for Cardiovascular Imaging and Great Ormond Street Hospital for Children (GOSH), London, WC1N 3JH, UK; 4 Department of Developmental Biology, MRC National Institute for Medical Research, The Ridgeway Mill Hill, London, NW7 1AA, UK; 5 Scuola Superiore Sant’Anna, Pisa and Fondazione “G. Monasterio” CNR - Regione Toscana, Pisa, 56124, Italy

**Keywords:** Cardiomyopathy, Heart failure, Trabeculation

## Abstract

**Background:**

Left ventricular noncompaction (LVNC) is a myocardial disorder characterized by excessive left ventricular (LV) trabeculae. Current methods for quantification of LV trabeculae have limitations. The aim of this study is to describe a novel technique for quantifying LV trabeculation using cardiovascular magnetic resonance (CMR) and fractal geometry. Observing that trabeculae appear complex and irregular, we hypothesize that measuring the fractal dimension (FD) of the endocardial border provides a quantitative parameter that can be used to distinguish normal from abnormal trabecular patterns.

**Methods:**

Fractal analysis is a method of quantifying complex geometric patterns in biological structures. The resulting FD is a unitless measure index of how completely the object fills space. FD increases with increased structural complexity. LV FD was measured using a box-counting method on CMR short-axis cine stacks. Three groups were studied: LVNC (defined by Jenni criteria), *n*=30(age 41±13; men, 16); healthy whites, *n*=75(age, 46±16; men, 36); healthy blacks, *n*=30(age, 40±11; men, 15).

**Results:**

In healthy volunteers FD varied in a characteristic pattern from base to apex along the LV. This pattern was altered in LVNC where apical FD were abnormally elevated. In healthy volunteers, blacks had higher FD than whites in the apical third of the LV (maximal apical FD: 1.253±0.005 vs. 1.235±0.004, p<0.01) (mean±s.e.m.). Comparing LVNC with healthy volunteers, maximal apical FD was higher in LVNC (1.392±0.010, p<0.00001). The fractal method was more accurate and reproducible (ICC, 0.97 and 0.96 for intra and inter-observer readings) than two other CMR criteria for LVNC (Petersen and Jacquier).

**Conclusions:**

FD is higher in LVNC patients compared to healthy volunteers and is higher in healthy blacks than in whites. Fractal analysis provides a quantitative measure of trabeculation and has high reproducibility and accuracy for LVNC diagnosis when compared to current CMR criteria.

## Background

Left ventricular noncompaction of the myocardium (LVNC) is a disorder of endomyocardial morphogenesis characterized by excessive left ventricular (LV) trabeculations and deep intertrabecular recesses.

LVNC occurs in association with many congenital cardiac disorders, but it is unclear whether isolated LVNC is a separate disease entity or a morphological trait shared by phenotypically distinct cardiomyopathies. Familial disease is estimated to occur in 18% to 64% of adults [1,2] with isolated LVNC, mostly as an autosomal dominant trait caused by mutations in many genes including those coding for sarcomere [3] and cytoskeletal proteins. Many patients with LVNC are completely asymptomatic, but some present with congestive heart failure, thromboembolism and arrhythmias, including sudden cardiac death [4,5].

Several attempts to quantify the extent of ventricular trabeculation as a means of diagnosing LVNC have been made using echocardiography but these criteria [1,6,7] poorly correlate with each other [8] and have poor reproducibility [9]. Even using high-contrast imaging modalities such as cardiovascular magnetic resonance (CMR) [10-12] and more recently computed tomography [13], there are still concerns over diagnostic accuracy [14,15].

Fine trabeculae are a feature of the normal LV and their extent may vary among healthy subjects of different racial backgrounds [8,14]. Existing criteria are problematic partly because they do not account for this normal spectrum of trabeculation. In addition some techniques are semi-quantitative [7], others rely on the subjective delineation of endocardial borders [11] or fail to consider the complex three-dimensional architecture of the myocardium [10]. The result is that there is no clear diagnostic standard.

A better approach may be to measure the complexity of the endocardial border, producing a continuous variable that can be used to both diagnose LVNC and assess its severity. One method to achieve this is to perform a fractal analysis of the endocardial border. Fractal analysis is a method of quantifying the complex geometric patterns which are encountered in biological, natural or mathematical structures [16,17]. The resulting unitless measure index is the fractal dimension (FD). It measures how completely the complex structure fills space. The range of possible FD for a fractal set is dictated by its topological dimension. For example, the endocardial borders visualized in two-dimensions, are more complicated than simple straight lines, so their FD must be > 1, but because they do not fill two-dimensional space completely, their FD must be < 2. Therefore the range of possible FD for an endocardial border is consistently a non-integer value, anywhere between 1 and 2.

In LVNC, the excessive trabeculae create a highly irregular endocardial border. Fractal analysis of these borders in LVNC should generate a higher FD when compared to normal hearts.

In this proof of principle study we have assessed the utility of measuring FD as a method for discriminating patients with LVNC from ethnically diverse healthy subjects. The specific aims were to assess: i) the FD in LVNC patients and healthy white and black volunteers and ii) the diagnostic accuracy and reproducibility of FD for identification of LVNC compared to current standard imaging criteria.

## Methods

### Study population

Three adult populations (aged 18–85 years) were studied between January 2010 and February 2012: LVNC cases (*n* = 30); healthy white (*n* = 75) and healthy black (*n* = 30) volunteers. Ethnicity was self-defined. Healthy normal volunteers had a body-mass index > 18.5 kg/m^2^ and < 30.0 kg/m^2^ and were not involved in high-level competitive sports.

LVNC cases (87% white; 13% black) were recruited from a dedicated cardiomyopathy clinic at The Heart Hospital, UCLH. Inclusion criteria for LVNC cases were: i) the fulfillment of Jenni *et al*’*s*. echocardiographic criteria for LVNC [6,18] and ii) the additional presence of at least one of the following: positive family history, associated neuromuscular disorder, regional wall motion abnormality, LVNC-related complications (arrhythmia, heart failure or thromboembolism) [10]. To ensure efficient segmentation of LV short-axis cines we excluded subjects with a high arrhythmogenic burden if this prohibited retrospective ECG gating for the duration of the scans. An ethics committee of the UK National Research Ethics Service approved the generic analysis of anonymized clinical scans. At the time of enrolment all participants gave written informed consent conforming to the declaration of Helsinki (V. revision, 2000).

### Cardiovascular magnetic resonance

Clinical scans (localizers, three long-axis views, black and white blood images, full LV short-axis stack) were performed on all subjects using a 1.5-T magnet (Avanto, Siemens Medical Solutions, Erlangen, Germany). CMR short-axis volumetric studies [19] were acquired from retrospectively-gated, breath-held, balanced, steady-state free-precession cines (slice thickness, 7.0 mm; inter-slice gap, 3.0 mm; flip angle, 70 - 80°; TR, 3.0 ms; TE, 1.33 ms; FOV read, 380 mm; FOV phase, 75%; sampled matrix resolution, 320 × 154; lines per segment, 12).

### Non-fractal image analysis

LV volumes, ejection fraction and LV mass were determined in all study participants using CMRtools (CVIS, London, UK). Two CMR methods for identifying LVNC were assessed in this study.

For the identification of LVNC using the Petersen method [10], all segments (excluding segment 17) were qualitatively assessed on each of the three long-axis cines for the presence of a distinct two-layered appearance. The noncompacted to compacted (NC/C) wall thickness ratio was calculated on the most trabeculated segment in each long-axis view and the maximal value was used for analysis. A ratio > 2.3 in diastole was considered diagnostic for LVNC.

For the Jacquier method [11], the epicardial contour was outlined on each end-diastolic short-axis frame. To measure the compacted LV mass, the endocardial border was drawn to include papillary muscle and exclude LV trabeculation. When the papillary muscles could not be clearly distinguished from the trabeculae, they were treated as trabeculae [20]. For total (compacted + noncompacted) LV mass, the endocardial border included both papillary muscles and LV trabeculation. Trabeculated LV mass was calculated as compacted LV mass subtracted from total LV mass with a value of trabeculated LV mass above 20% of the total LV mass considered diagnostic for LVNC.

### Fractal analysis

**Figure 1:**
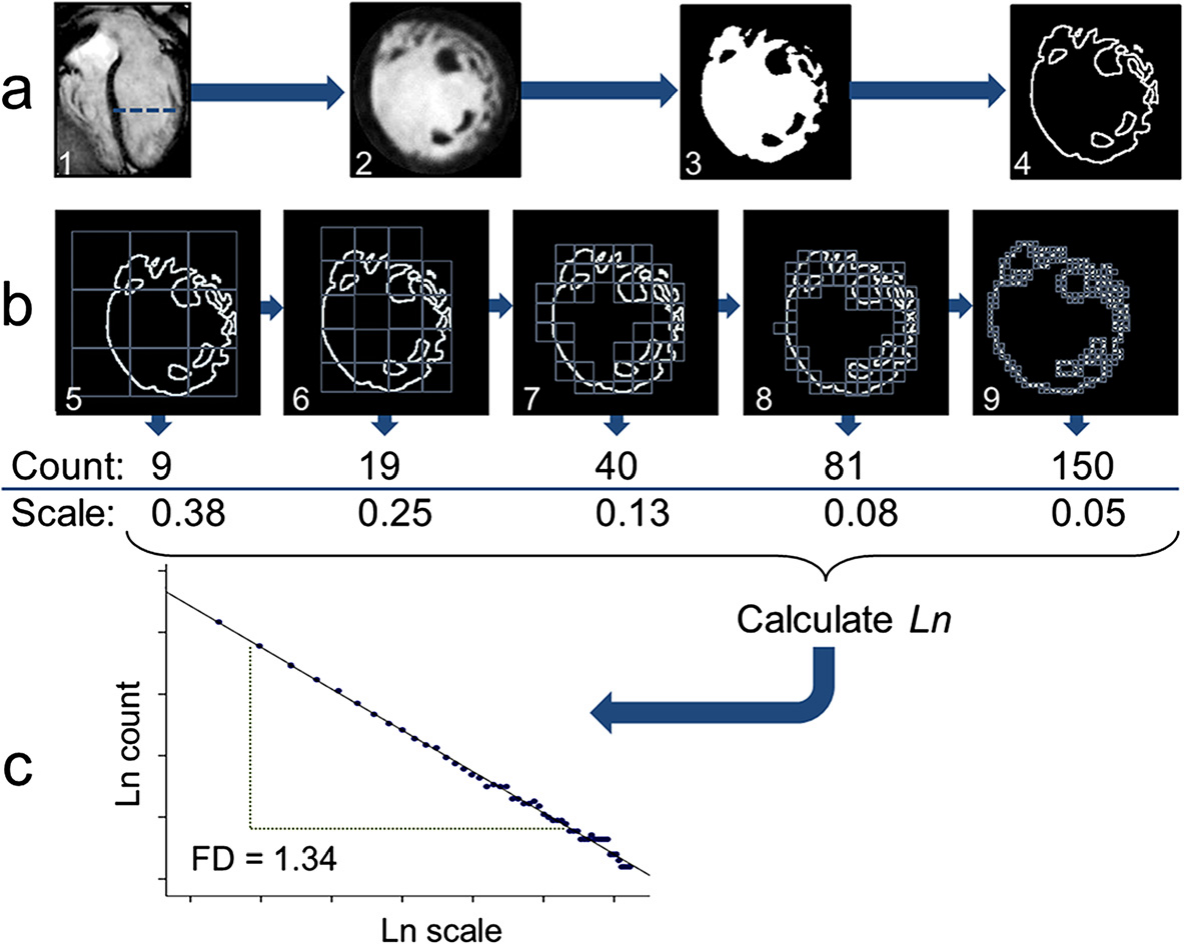
**Image processing sequence and fractal analysis of left ventricular cine images.** Example analysis of a single slice (**a**-2) out of a cine volumetric left ventricular stack, belonging to an LVNC case. Dashed line across the 4-chamber view marks the slice location (**a**-1). Automatic thresholding, binarization (**a**-3) and edge-detection (**a**-4) are followed by fractal analysis (**b**). In the box-counting method a series of grids of boxes of progressively smaller size are laid over the ROI and boxes containing detail are counted (**b**-5 to 9). The same set of grid calibres is applied to the ROI in four different orientations. In this pictorial representation, only 5 box sizes are shown but the complete analysis for this slice actually involves 55 box sizes. Each orientation generates a separate natural logarithmic plot of box-count (*y* axis) against scale (*x* axis, calculated from box/image size) (**c**). The slope of the line-of-best-fit across the points represents a FD. The mean value from the four plots is the slice FD. In this case, the straight line supports the existence of a fractal pattern. FD for this slice is 1.34. FD = fractal dimension; Ln = natural logarithm; LVNC = left ventricular noncompaction; ROI = region of interest.

Fractal analysis was performed on the end-diastolic frames of each short-axis slice in the LV stack using an in-house macro designed for public domain software, ImageJ [21] (version 1.38×, National Institute of Health, Bethesda, MD, USA). Analysis was divided into two parts: image segmentation to extract the endocardial border and then calculation of the FD of the endocardial border using the box-counting method. The first stage of image segmentation was thresholding and binarization of the LV blood pool and myocardium (Figure 1a). To ensure standardized treatment, this was done using an automated thresholding technique (a variation of the iterative intermeans IsoData algorithm) [22]. The endocardial border of the binarized image was then extracted by computing edges in the areas of highest gradient magnitude using the Sobel operator [23]. Papillary muscles and parts of the subvalvular apparatus, which were extracted at the time of segmentation, also provided edges to the final image submitted to fractal analysis.

Fractal analysis of the endocardial border was performed using the standard box-counting method available in the FracLac plug-in (Karperien, A., version 2.5, 1e). In this method, a grid of known spacing (scale) was placed over the endocardial border image (border pixels = 1, background pixels = 0) and the number of boxes that contained non-zero pixels was counted (Figure 1b). This process was then repeated for multiple grids with increasing spacing. As the scale increases, the number of boxes containing the object decreases exponentially and the exponent is equivalent to the FD. To quantify the exponent, natural logarithmic plots of the number of boxes against scale and the gradient (−FD) was estimated using linear regression (Figure 1c). In our method, the maximum spacing was set to 45% of the diameter of the endocardial border and the minimum box size was two pixels.

To assess the global LV FD, the FD from each slice in the LV were averaged. To assess local fractal characteristics, the maximal FD in the basal, mid and apical thirds of the LV were recorded.

The endocardial borders generated during fractal analysis were also used to create a simpler semi-automated measure of endocardial complexity, which involved calculating the perimeter of each endocardial border image. Summing the perimeter of each slice gave the total heart endocardial perimeter, which was indexed to body-surface area.

### Statistical analysis

A two-sided p value < 0.05 was considered significant. Statistical analysis was performed in SPSS for Windows version 20.0 (Chicago, IL, USA). Descriptive data are expressed as mean ± standard error of mean (s.e.m.) except where otherwise stated. Distribution of data was assessed on histograms and using Shapiro-Wilk test. Patient group characteristics were non-normally distributed and compared by the Kruskal-Wallis and Pearson’s Chi Square non parametric tests as appropriate. Correlation between continuous variables was assessed with Pearson’s Correlation Coefficient. FD was compared between the three study populations using Student’s *t*-Test. For the fractal method and perimetry, area under the receiver operating characteristics (ROC) curve (AUC) was calculated using the method described by Hanley et al. [24]. Optimal threshold values for diagnosing LVNC were calculated as the Youden Index J, where J = max (sensitivity*c* + [specificity*c*-1]), with *c* representing ranges over all possible criterion values.

Intra and inter-observer reproducibility of fractal analysis, perimetry, Petersen and Jacquier techniques were assessed on 60 subjects (*n* = 20 for each of LVNC cases, healthy white and healthy black volunteers) by two experienced and blinded readers (reader G.C. for intra-observer and reader A.B. for inter-observer ratings). Intra-observer readings were performed with one-month temporal separation between repeat analyses. Paired measurements from fractal analysis were evaluated using the Bland-Altman method [25] and by calculating coefficients of variation and repeatability coefficients (RC), where RC = 1.96 √(ΣDi^2^/*n*-1) (Di being the absolute difference between repeat measurements and *n*, the number of measurements). Intraclass correlation coefficient (ICC) was used to compare reproducibility between the various methods. Variability of binary outcome data for each method using established cut-off values was assessed using Fleiss’ Kappa test for concordance. For all methods, sensitivity, specificity, positive and negative predictive values with exact 95% CI are reported [26].

## Results

### Demographics

All three groups were well matched in terms of age, gender and body characteristics (Table 1). Clinical characteristics of LVNC cases are summarized in Additional file 1: Table S1. There was no difference in LV volumes or function between the healthy white and black populations. LVNC patients had larger LV volumes and lower ejection fractions than healthy volunteers.

**Table 1:** **Clinical characteristics of the 135 subjects**, **by population studied**

**Variable**	**LVNC Cases**	**Healthy whites**	**Healthy blacks**
	**(*n *30= , ± s**.**d.)**	**(*n *= 75, ± s.d.)**	**(*n *= 30, ± s.d.)**
Age (yrs)	41 ± 13	46 ± 16 (p = NS)	40 ± 11 (p = NS)
Male/Female	16/14	36/39 (p = NS)	15/15 (p = NS)
Weight (Kg)	80 ± 14	77 ± 14 (p = NS)	78 ± 19 (p = NS)
Height (cm)	172 ± 6	171 ± 11(p = NS)	169 ± 9 (p = NS)
BSA (m^2^)	1.9 ± 0.04	1.9 ± 0.03 (p = NS)	1.9 ± 0.05 (p = NS)
BMI (kg/m^2^)	27 ± 4	26 ± 4 (p = NS)	27 ± 6 (p = NS)
LVEDV*i* (mls/m^2^)	104 ± 49	74 ± 16 (p < 0.01)	73 ± 13 (p < 0.01)
EF (%)	52 ± 17	69 ± 4 (p < 0.001)	72 ± 8 (p < 0.001)
Max. Apical FD	1.392 ± 0.053	1.235 ± 0.03 (p < 0.00001*)	1.253 ± 0.025 (p < 0.00001†)
**Variable**	**LVNC Cases**	**Healthy Whites**	**Healthy Blacks**
	(***n*** = **20**, ± **s**.**d**.)	(***n*** = **20**, ± **s**.**d**.)	(***n*** = **20**, ± **s**.**d**.)
NC/C Ratio‡	2.87 ± 0.94	1.37 ± 0.40 (p < 0.00001)	1.38 ± 0.48 (p < 0.00001)
Trab. Mass (% LV)§	33.35 ± 8.83	18.12 ± 5.72 (p < 0.00001)	20.05 ± 7.43 (p < 0.00001)

* Two-tailed p value for *t*, comparing FD between LVNC cases and healthy whites.

† Two-tailed p value for *t*, comparing FD between LVNC cases and healthy blacks.

‡ Results from the Petersen analysis. Two-tailed p value for *t,* comparing end-diastolic NC/C ratios between healthy volunteers and LVNC.

§ Results from the Jacquier analysis. Two-tailed p value for *t,* comparing trabeculated mass (% of LV) between healthy volunteers and LVNC.

BSA = body-surface area; BMI = body-mass index; C = compacted; EF = ejection fraction; FD = fractal dimension; LVED*i* = left ventricular end-diastolic volume indexed to BSA; LVNC = left ventricular noncompaction; max. = maximal; NC = noncompacted; NS = not significant (after comparison with LVNC cases); s.d. = standard deviation; Trab. = trabeculated; Yrs = years.

### Fractal dimension

**Figure 2:**
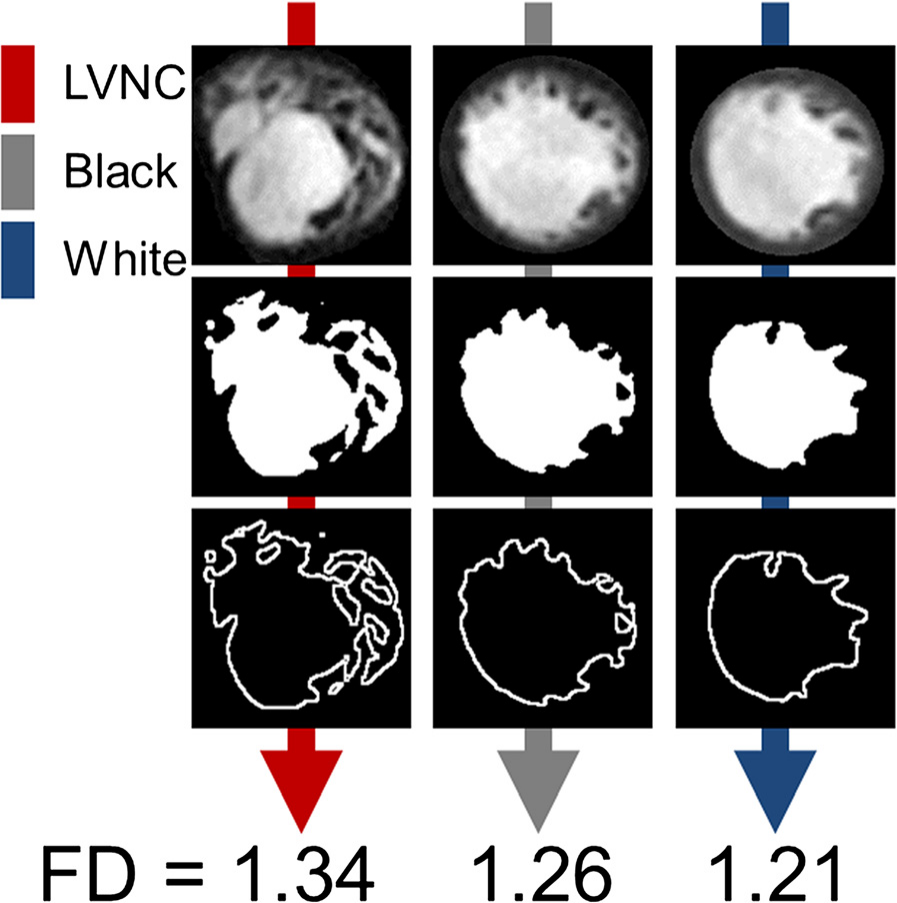
**Illustrative left ventricular slices from each of the three study populations with corresponding FDs.** Fractal analysis of each slice generates a value for the FD. In this study we demonstrate that FD differs significantly between LVNC, healthy black and white populations. Abbreviations as in Figure 1.

**Figure 3:**
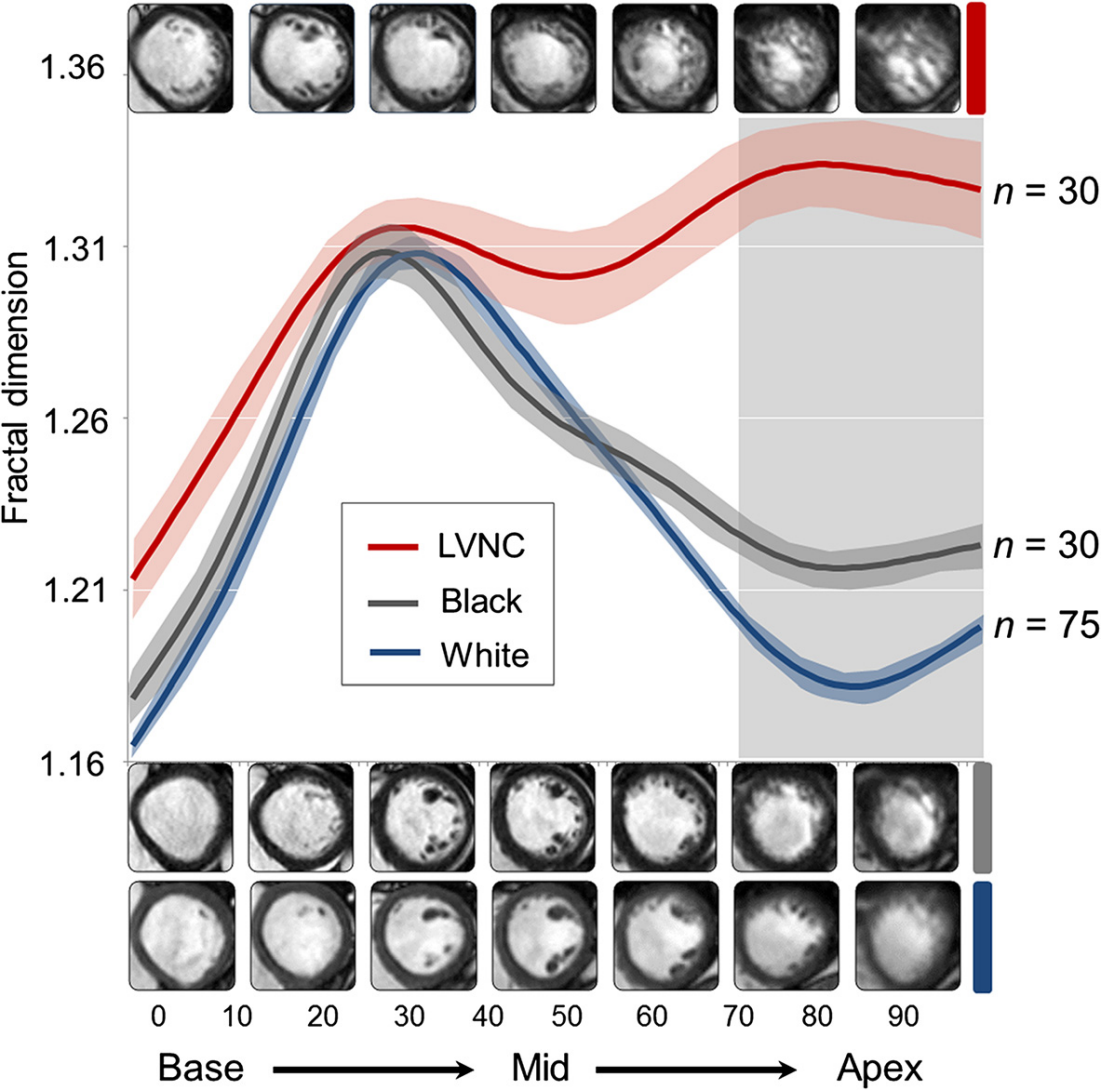
**FD across the left ventricle from base to apex.** This interpolated dataset summarizes regional differences in left ventricular trabecular complexity. Population mean FD (solid lines) and s.e.m. (shaded areas) are shown. The maximum difference between groups was observed in the apical third of the ventricle (vertical grey bar). Illustrative end-diastolic CMR slices are shown for each of the three populations. CMR = cardiovascular magnetic resonance; s.e.m. = standard error of mean; other abbreviations as in Figure 1.

Fractal analysis could be performed in all hearts with an average analysis time of 5.28 ± 0.41 minutes (± s.d.) (Figures 2 and 3). Global LV FD was greater in LVNC patients (1.290 ± 0.008) compared to healthy white (1.228 ± 0.002; p < 0.00001) and healthy black volunteers (1.246 ± 0.005; p < 0.0001). Blacks had higher global LV FD than whites (p < 0.01). In all populations the lowest FD across the LV was registered in the basal slice above the level of any papillary muscles or subvalvular apparatus (LVNC, 1.213 ± 0.012; healthy white, 1.164 ± 0.003; healthy black, 1.179 ± 0.008).

**Figure 4:**
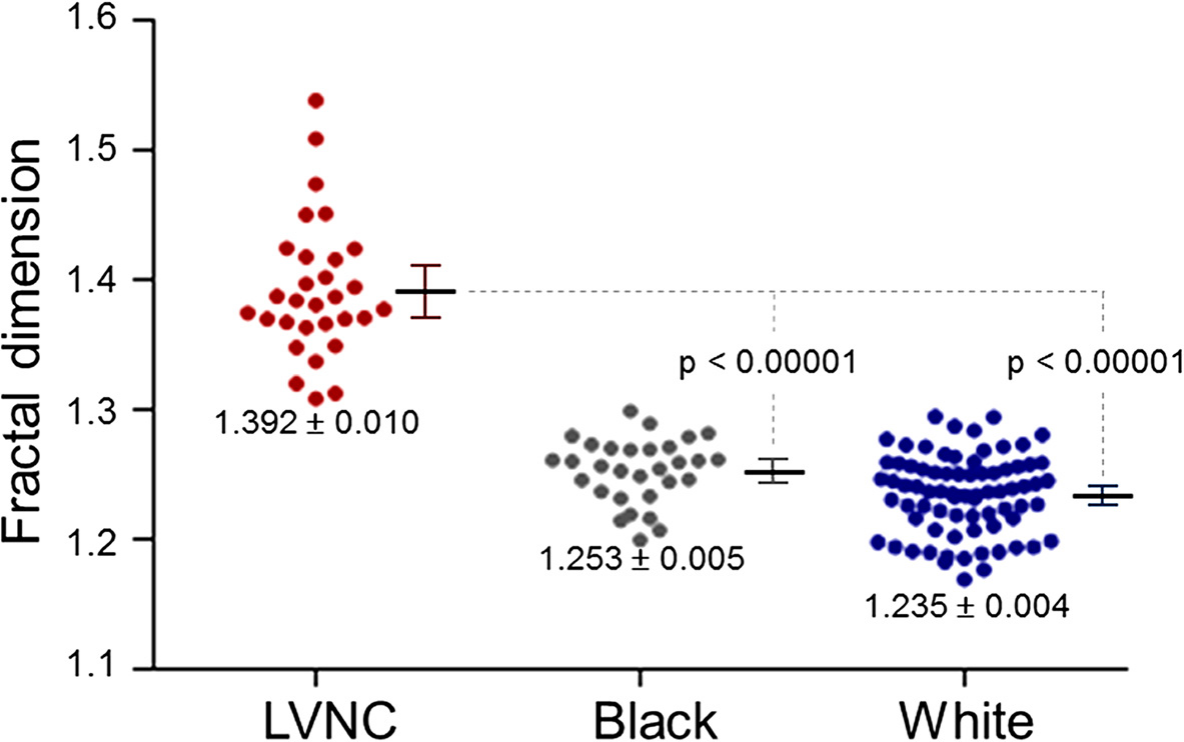
**Maximal apical FD in all subjects studied.** Scatter plots show the distribution of FD in the apical third of the ventricle for all subjects studied. Error bars representing mean values (thick black lines) and upper and lower 95% CI (whiskers) are also shown. There was no overlap in maximal apical FD: no LVNC patient had a maximal apical FD < 1.309 (*n* = 30); all healthy whites had a maximal apical FD < 1.295 (*n* = 75) and all healthy blacks had a maximal apical FD < 1.299 (*n* = 30). CI = confidence interval; other abbreviations as in Figure 1.

In the basal third of the LV, there was no difference in maximal FD between LVNC patients (1.325 ± 0.007) and healthy black (1.322 ± 0.007, p = 0.76) or white (1.317 ± 0.004, p = 0.35) volunteers. In the middle third, maximal FD in LVNC (1.348 ± 0.011) was higher than in healthy black and white populations (1.311 ± 0.008 and 1.318 ± 0.004 respectively, p < 0.05 both). The greatest difference in maximal FD between LVNC and healthy populations was registered in the apical third (p < 0.00001). Maximal apical FD was: LVNC, 1.392 ± 0.010; healthy white, 1.235 ± 0.004; healthy black, 1.253 ± 0.005 (Figure 4). At this location healthy blacks had higher maximal FD than healthy whites (p < 0.01). There was no overlap in maximal apical FD between LVNC and healthy volunteers.

No significant correlation was demonstrated between FD and body-surface area in the three populations (LVNC: *r* = −0.10, p = 0.61; healthy white: *r* = 0.2, p = 0.10; healthy black: *r* = 0.08, p = 0.70). In the LVNC group but not in the healthy volunteers, a correlation existed between FD and LV size (LVNC: *r* = 0.5, p < 0.01; healthy white: *r* = 0.12, p = 0.34, healthy black: *r* = 0.002, p = 0.99). FD remained elevated even in those LVNC patients without dilatation (59% of LVNC cohort) when compared to healthy volunteers (p < 0.0001). No significant correlation was demonstrated between FD and ejection fraction in the three populations (LVNC: *r* = −0.31, p = 0.09; healthy white: *r* = 0.10, p = 0.43; healthy black: *r* = 0.29, p = 0.16).

### Diagnostic accuracy

In patients with LVNC, NC/C ratios measured using the Petersen method and trabeculated mass (% of LV) measured using the Jacquier technique, were significantly higher than in healthy volunteers (Table 1).

**Figure 5:**
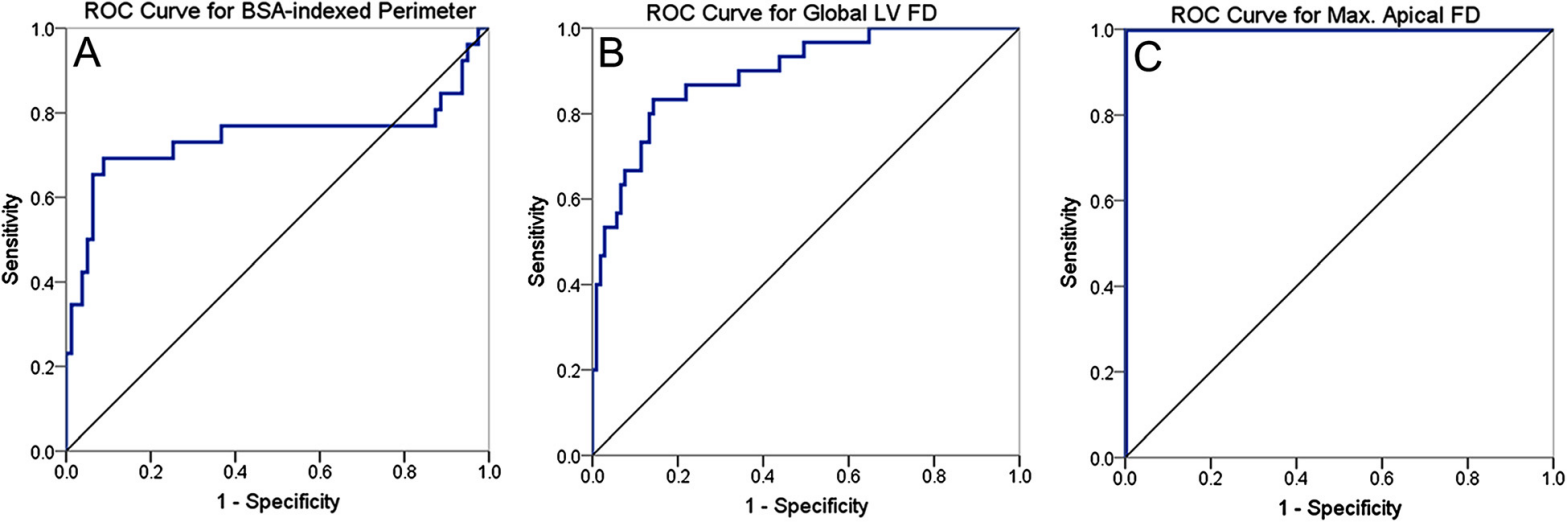
**ROC curves for perimetry and the fractal method.** ROC curves (in blue) describing the performance of BSA-indexed perimetry (**A**), global LV FD (**B**) and maximal apical FD (**C**) for LVNC diagnoses using as a reference, patient classification according to our study criteria for inclusion of LVNC cases. Diagonal reference lines (in black) are also shown. In white and black populations: BSA-indexed perimetry ≥ 3252 mm/m^2^ predicts LVNC with specificity 91% and sensitivity 70% (AUC ROC curve 0.741, CI 0.60 – 0.89); global LV FD ≥ 1.26 predicts LVNC with specificity 86% and sensitivity 83% (AUC ROC curve 0.893, CI 0.83 – 0.96); maximal apical FD ≥ 1.30 predicts LVNC with specificity and sensitivity 100% (AUC ROC curve 1.0). ROC = receiver operating characteristics; AUC = area under the curve; BSA = body-surface area; max. = maximum; other abbreviations as in Figures 1 and 4.

On ROC analysis, the optimum diagnostic threshold for global LV FD was 1.26, for maximal apical FD it was 1.30 and for total LV perimeter it was 3252 mm/m^2^ (Figure 5). For fractal analysis and perimetry, sensitivity, specificity, positive and negative predictive values were calculated using ROC-derived thresholds while for Jacquier and Petersen methods, previously published diagnostic cut-offs were used. Maximal apical FD discriminated LVNC from healthy volunteers with no overlap in FD. Of the 20 LVNC cases, 6 were mislabelled as normal by the Petersen method. Of the 40 healthy volunteers, 19 were mislabelled as having trabeculation in the LVNC range by the Jacquier approach. Accuracy statistics for all methods are summarized in Table 2.

**Table 2:** **Accuracy statistics for the fractal method and comparator CMR criteria**.

Accuracy statistic*	**Max. Apical FD**	**Global LV FD**	**Perimetry**	**Petersen**	**Jacquier**
Sensitivity (95% CI, UL-LL)	100 (88–100)	83 (61–92)	70 (48–85)	70 (46–87)	95 (73–100)
Specificity (95% CI, UL-LL)	100 (97–100)	86 (78–92)	91 (86–97)	98 (85–100)	53 (36–68)
PPV (95% CI, UL-LL)	100 (88–100)	63 (46–78)	72 (50–87)	93 (66–100)	50 (34–66)
NPV (95% CI, UL-LL)	100 (97–100)	94 (86–97)	92 (85–96)	87 (72–94)	95 (75–100)

*Sensitivity, specificity, PPV and NPV values are quoted as %.

CI = confidence interval; LL = lower limit; NPV = negative predictive value; PPV = positive predictive value; UL = upper limit; other abbreviations as in Table 1.

### Reproducibility

The fractal method showed good reproducibility without bias on Bland Altman plots, and produced small RC (intra-observer, 0.059; inter-observer, 0.067) and favourable coefficients of variation (5% for both intra and inter-observer readings). Comparison between methods showed better reproducibility for global LV FD, maximal apical FD and total LV perimetry when compared to the Petersen and Jacquier methods (Table 3).

**Table 3:** Summary of reproducibility testing

**Method†**	**Intra-observer κ**			**Inter-observer κ**			**Intra-observer ICC**			**Inter-observer ICC**		
	**κ**	**95% CI**		**κ**	**95% CI**		**ICC**	**95% CI**		**ICC**	**95% CI**	
		**LL**	**UL**		**LL**	**UL**		**LL**	**UL**		**LL**	**UL**
Max. Apical FD	0.9***	0.7	1.2	0.9***	0.7	1.2	0.97	0.95	0.98	0.96	0.93	0.97
Global LV FD	0.9***	0.6	1.2	0.9***	0.6	1.1	0.98	0.97	0.99	0.97	0.95	0.98
Perimetry	0.9***	0.6	1.2	0.9***	0.7	1.3	0.98	0.97	0.99	0.98	0.96	0.99
Petersen	0.8**	0.5	1.0	0.7**	0.4	0.9	0.89	0.82	0.93	0.83	0.73	0.89
Jacquier	0.5*	0.2	0.8	0.5*	0.3	0.9	0.86	0.87	0.95	0.89	0.82	0.93

*** Excellent agreement between ratings, κ of > 0.8.

** Strong agreement, κ of 0.61 – 0.8.

* Moderate agreement, κ of 0.41 – 0.6.

† For maximal apical FD, global LV FD and perimetry, cut-off values for LVNC diagnoses were derived from ROC analysis. For Petersen and Jacquier techniques, cut-off values for LVNC diagnoses were derived from previously published thresholds.

ICC = intraclass correlation coefficient; κ = Fleiss’ kappa; ROC = receiver operating characteristics; other abbreviations as in Tables 1 and 2.

## Discussion

The main findings of this study were that: i) FD was significantly higher in LVNC patients compared to healthy volunteers, ii) FD was higher in healthy blacks than in whites, and iii) fractal analysis was accurate and reproducible when compared to current techniques. The fractal method permits better identification of patients with LVNC.

### The fractal dimension

Although classically used to describe ‘pure fractals’ (objects that are self-similar at infinite scales), fractal analysis can also be performed on real-world objects [27]. In nature, these include river networks, leaf structures and vascular trees with a higher FD suggesting more complexity (in our case, endocardial borders in LVNC cases were more irregular and produced higher FD on account of the hypertrabeculation).

Fractal analysis has already found widespread application in the medical imaging field for the analysis of lungs [28], vasculature [29,30], bone [31] and brain [27] and across several imaging modalities (plain radiographs, retinal photography, computed tomography, magnetic resonance imaging).

### Distribution of trabeculation

The elevated FD noted at the mid-ventricular level in both LVNC and healthy volunteers (Figure 3) is explained by the presence here of the papillary muscles and subvalvular apparatus - a constant feature of both healthy and noncompacted hearts. This is in line with previously reported data [32-34].

While the apex of healthy hearts is known to be lined by numerous trabeculae [35,36], these parietal structures are fine [37] and with our resolution their segmentation approximates the true complexity, in much the same way as their measured height for NC/C calculations or their traced contours for trabeculated mass %, approximates the ground truth towards the apex. This is not so in LVNC where trabeculae are larger and easier to segment producing the highest apical FD.

### Effect of ethnicity on trabeculation

The healthy LV is more trabeculated in blacks than in whites as evidenced by higher global LV FD and maximal apical FD in this study. This ethnic difference, suggested before [8,14,38] but never quantitated, remains unaccounted for by current imaging criteria.

In a large multi-ethnic cohort free from hypertension and cardiac disease at baseline (*n* = 367) Kawel [14] reported that 43% had a noncompacted to compacted wall thickness ratio of > 2.3 pointing towards oversensitivity of the Petersen method. This finding was not borne out in our study. The reasons for this could be several: firstly Kawel studied a substantially larger and more ethnically-diverse population (that also included Chinese subjects); secondly we, like Petersen, employed a single Siemens scanner together with CMRtools software for all our analysis; thirdly, the Bethesda group incorporated into their analyses two additional methodological steps (cross-referencing long-axis datasets to the short-axis cines before recording measurements and correcting for chemical shift artefact at the epicardial border) that were not part of the original Petersen description. The cumulative influence of these factors on the sensitivity of the method are not known.

### Diagnostic power

Accurate diagnosis of LVNC is required for the appropriate management of confirmed cases and it avoids the unjustified diagnosis of cardiomyopathy in healthy subjects [39,40]. Applied singly, current criteria are shown to be moderately accurate [14]. Their diagnostic performance may be improved by applying a more complex, combined criteria approach involving several CMR parameters [12]. In this feasibility study, we compared healthy hearts to a group of clinically and morphologically severe LVNCs and we show how the maximal apical FD may serve as a useful diagnostic marker for LVNC. Global LV FD also has reasonable diagnostic accuracy, although it doesn’t significantly outperform either LV perimetry or the Petersen methods. This is because global LV FD is an average measure and includes fractal values from the basal and mid-ventricular levels where LVNC cases are not too dissimilar from healthy volunteers. This has a tendency to attenuate the larger apical differences in FD. Nevertheless, global LV FD has a greater AUC than LV perimetry, suggesting that the extra geometric information encoded in the FD is of diagnostic benefit. Both fractal methods and perimetry demonstrated very high levels of reproducibility when compared to other methods mainly because they were semi-automated rather than investigator-dependent techniques. CMR fractal analysis may be considered to be a semi-automated technique because user-interaction is limited to ROI delineation.

### Effect of left ventricular size on fractal dimension

We found a positive correlation between FD and LV size within the LVNC population. This raises the question about whether the higher FD recorded in LVNC cases could simply have been the result of larger cardiac size. To address this, we showed how FD remained elevated even amongst those LVNC cases with non-dilated ventricles. Furthermore, the box-counting approach is scaled so each FD is calculated relative to the size of the ROI (size of the LV). This correlation between FD and LV size is interesting, and merits further study involving a larger more extensively-phenotyped population.

### Limitations

The main limitation of this study is the lack of an LVNC reference-standard, so a composite diagnostic criterion was used to define our LVNC cases. This was a single-centre study and thus the sensitivity of the developed methodology to the use of different scanners, different manufacturers and to varying scan parameters has not been tested. The last apical slice of short-axis stack, containing a few milliliters of blood and highly prone to partial voluming, was omitted from analysis (in line with the approaches of Petersen et al. [10] and Kawel et al. [14]). Further work would be needed with this (and other) technique/s to determine the effects of image ascertainment on measurements. The impact of noise, high arrhythmogenic burden and presence of contrast on the fractal analysis will need to be examined. Future research should clarify whether global or region-specific FD is better and under which circumstances. Fractal properties of other cardiac phenotypes where trabeculae are also believed to be abnormal such as hypertrophic cardiomyopathy, dilated cardiomyopathy or congenital heart disease will need to be explored. A fractal-based definition of ventricular trabeculation could serve as a useful adjunct for the improved diagnosis of LVNC but it describes only part of the phenotype – other features remain important: thinning of the compacted wall [41] (which fractal analysis does not consider), scarring, dilatation, LV function, thrombi, family history, genetics, arrhythmia and associated neuromuscular disease.

The LV FD could permit the stratification of hearts imaged by CMR across a spectrum of normal to abnormal trabecular patterning but ethnically-appropriate, population-based, normative reference ranges for trabeculation in health will be needed for this.

## Conclusions

In this study, we describe a novel reproducible approach to measuring LV trabecular complexity using fractal analysis applied to CMR. FD is higher in LVNC patients compared to healthy volunteers and is higher in healthy blacks than in whites. In subjects with a high pre-test probability for disease, the biological signal measured by fractal analysis distinguishes LVNC from health with high diagnostic accuracy.

## Abbreviations

AUC: Area under curve; CI: Confidence interval; CMR: Cardiovascular magnetic resonance; FD: Fractal dimension; ICC: Intraclass correlation coefficient; LV: Left ventricle/ventricular; LVNC: Left ventricular noncompaction; NC/C: Noncompacted to compacted ratio; RC: Repeatability coefficient; ROC: Receiver operating characteristics; ROI: Region of interest; s.d.: Standard deviation; s.e.m: Standard error of mean.

## Competing interests

The authors declare that they have no competing interests.

## Authors’ contributions

All authors have contributed significantly to the submitted work: JCM. and PME conceived and directed the project. JCM, VM, PME and GC wrote the article. JCM, VM, PME, ASF and GC developed the fractal technique for use with CMR. GC, CC, ASF, DS, SA and JCM performed the CMR imaging in humans. GC and JCM analyzed the human data. GC and AB performed the reproducibility experiments. PME, VM, TM, RW and WJM provided expert advice and critical review of the manuscript. All authors read and approved the final manuscript.

## Supplementary Material

Additional file 1: Table S1 Detailed clinical characteristics of the 30 LVNC cases. Click here for file
