# Supplementary material for: Quantification of left ventricular trabeculae using fractal analysis
Source: J Cardiovasc Magn Reson. 2013 May 10;15(1):36. doi: 10.1186/1532-429X-15-36 (PMC3680331; doi:10.1186/1532-429X-15-36)
Supplement: Additional file 1: Table S1 — Detailed clinical characteristics of the 30 LVNC cases. [file 1532-429X-15-36-S1.doc]

**Supplemental Table 1: Detailed clinical characteristics of the 30 LVNC cases.**

| Cases | Age/Dg | Gender | Ethnicity | ECG | NYHA | Arrhythmia | FH | WMA | EF | NMD | CMR Slices |
| --- | --- | --- | --- | --- | --- | --- | --- | --- | --- | --- | --- |
| 1 | 41/34 | M | W | - | 1 | - | + | - | 70 | - | 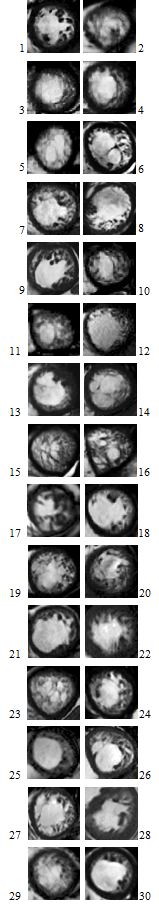 |
| 2 | 70/66 | F | W | + | 2 | + | - | - | 78 | + |
| 3 | 40/36 | F | B | - | 2 | - | + | + | 62 | + |
| 4 | 33/29 | M | W | - | 1 | - | - | + | 45 | - |
| 5 | 29/22 | F | W | + | 1 | - | + | + | 66 | - |
| 6 | 78/74 | F | W | + | 1 | + | + | + | 33 | - |
| 7 | 31/30 | M | W | + | 1 | - | - | + | 45 | - |
| 8 | 56/52 | F | W | + | 2 | + | - | + | 18 | - |
| 9 | 53/50 | M | W | + | 1 | + | - | + | 40 | - |
| 10 | 60/60 | F | W | + | 2 | - | - | + | 45 | - |
| 11 | 41/40 | F | W | + | 3 | + | + | + | 45 | + |
| 12 | 53/50 | M | B | + | 2 | - | - | + | 20 | - |
| 13 | 30/30 | M | W | + | 1 | + | - | - | 47 | + |
| 14 | 49/48 | M | W | + | 3 | + | - | - | 38 | - |
| 15 | 46/35 | F | W | - | 1 | + | - | - | 77 | + |
| 16 | 65/53 | F | W | + | 2 | + | - | + | 54 | + |
| 17 | 39/35 | M | W | + | 2 | + | - | - | 60 | - |
| 18 | 67/65 | F | W | + | 1 | + | - | - | 77 | - |
| 19 | 66/63 | F | W | + | 3 | + | - | + | 29 | - |
| 20 | 33/29 | M | W | + | 1 | + | - | - | 49 | + |
| 21 | 36/35 | M | W | - | 2 | + | + | - | 53 | - |
| 22 | 40/20 | M | W | + | 1 | - | - | + | 50 | - |
| 23 | 24/20 | M | W | + | 1 | - | + | - | 77 | - |
| 24 | 27/20 | F | B | - | 1 | - | + | - | 61 | - |
| 25 | 52/33 | F | W | - | 4 | + | + | + | 73 | - |
| 26 | 43/33 | M | W | - | 2 | + | + | + | 61 | - |
| 27 | 75/75 | M | W | + | 3 | + | - | + | 32 | - |
| 28 | 37/13 | M | W | + | 1 | + | - | + | 71 | - |
| 29 | 68/66 | M | W | + | 2 | + | - | + | 45 | - |
| 30 | 37/33 | F | B | + | 1 | + | - | - | 67 | - |

Characteristics of the LVNC population focussing on the presence of factors influencing the pre-test probability for disease. Left ventricular short-axis CMR slices for each of the study participants (1-30) are presented on the right.

Age/Dg = age in years/age at time of LVNC diagnosis; B = self-reported black ethnicity; ECG = electrocardiogram (- normal; + abnormal); EF = ejection fraction (%); F = female gender; NYHA = New York Heart Association functional class (1- 4); FH = family history for LVNC (- none; + present); M = male gender; NMD = neuromuscular disease (- none; + present); W = self-reported white ethnicity; WMA = wall motion abnormality (- none; + present).
